# In Vitro Efficacy of Domestic Techniques for Disinfection of Toothbrushes Contaminated With *Enterococcus faecalis*

**DOI:** 10.1155/2024/3509832

**Published:** 2024-10-18

**Authors:** Gina Alessandra Donayre-Salvatierra, Julissa Amparo Dulanto-Vargas, Raul M. Olaechea, Oscar Reátegui, Kilder Maynor Carranza-Samanez

**Affiliations:** ^1^School of Dentistry, Universidad Científica del Sur, Lima, Peru; ^2^Research Group in Dental Sciences, School of Dentistry, Universidad Científica del Sur, Lima, Peru; ^3^Microbiology and Genomics Research Laboratory, Universidad Científica del Sur, Lima, Peru; ^4^Research Group, Characterization, Transformation and Sustainability of the Natural Resources of Peru (CTS Group), Universidad Científica del Sur, Lima, Peru

**Keywords:** *Allium sativum*, disinfection, microwave, toothbrush, vinegar

## Abstract

**Introduction:** Oral hygiene education for patients is fundamental in preventive-promotional dentistry. The disinfection of toothbrushes (TBs) must be integrated into this context due to their proximity to contaminant sources that make them vulnerable to cross infection in homes. The objective of this study was to compare the efficacy of domestic techniques for disinfection of TBs.

**Materials and Methods:** We performed an in vitro study of 76 TBs contaminated with *Enterococcus faecalis* (*Ef*) ATCC 29212 subjected to different disinfection protocols: G1. Distilled water (DW; negative control; *n* = 8), G2. Dimethylsulfoxide (DMSO)10% (negative control; *n* = 8), G3. Chlorhexidine (CHX) 0.12% (positive control; *n* = 15), G4. 100% white vinegar (WV; *n* = 15), G5. Microwave (MW) at 700 W (*n* = 15), and G6. 200 mg/mL of certified alcoholic extract of purple garlic (GARLIC) from Arequipa (*Allium sativum L*; *n* = 15). Bacterial count was assessed by colony-forming units (CFU/mL) categorized as contamination: low (<30), medium (30–300), and high (>300). The Kruskal–Wallis test with post hoc pairs was used at a significance level of *p* < 0.05.

**Results:** Efficacy against *Ef* showed highly significant differences between groups (*p* < 0.001) with lower median CFU/mL in G3 and G4 (Me = 0 [IQR (interquartile range) = 0]: low) and G5 (Me = 6000 [IQR = 45,000]: low/medium) versus negative controls (Me = 378,500 and 5,020,000 [IQR = 4,605,000 and 6,760,000]: medium/high; *p* ≤ 0.019). The counts of the G5 were not statistically different than G3, G4, and G6 (*p* > 0.06). The G6 (Me = 1,510,000 [IQR = 590,000]: medium) was inferior to G3 and G4 (*p* < 0.001), but similar to both negative control groups (*p* > 0.999).

**Conclusions:** Disinfection of TBs with CHX, WV, and MWs produces a significant effective reduction in the count of *Ef*.

## 1. Introduction

Oral health is fundamental to overall well-being [1], since healthy dentition allows basic functioning, such as chewing, smiling, speaking, and socializing [2]. It is known that oral colonizing bacterial are associated with health and disease processes [3]. For this reason, oral hygiene is essential and is achieved by applying control and cleaning measures with mechanical and chemical methods [4, 5]. Among the different cleaning measures available, the toothbrush (TB) is the traditional element par excellence that has the greatest accessibility [6–8].

The TB is an oral hygiene tool used for the removal of biofilm, preventing dental caries, and periodontal disease. Nonetheless, disinfection of TBs is given little attention at home [8] despite their being cross contaminated by microorganisms in the bathroom that are the same as those that transmit diseases [1, 9–11]. According to the literature after the use of TBs, different microorganisms and other contaminants, such as biological fluids, oral waste, and toothpaste residues can remain on the TB [12, 13]. Microorganisms are derived from the oral or external environment [9] and are able to lodge on the surfaces of the bristles, forming a habitat for bacterial survival, especially in retentive areas [14].

The risk of TB contamination increases with prolonged and/or repetitive use and incorrect storage [15]. TB cleanliness has been analyzed in studies with chemical agents (chlorhexidine [CHX] [1, 5, 8, 11, 15–24], cetylpyridiniumchloride [11, 18], triclosan [20]), disinfectants (white vinegar [WV] [1, 10, 16, 17, 21], hydrogen peroxide [16]), sodium hypochlorite [1, 8, 10, 16, 17], povidone-iodine [15, 16], alcohol [16, 17, 22]), radiation (ultraviolect rays [10, 15, 16, 18], microwave [MW] [10, 16, 20, 24, 25]), and natural agents (garlic and green tea and tea tree oil [18, 23])). A systematic review found that the reduction of bacterial load on TBs was effective with radiation and natural agents, but insignificant with CHX [26].

The formation of a reservoir of pathogens is more sensitive of greater concern in people with severe or systemic oral diseases [8, 12, 16, 27]. This is due to the fact that injury to the gums during brushing can favor the entry of microorganisms into the bloodstream and even reinfection by viruses, thereby being considered in this context as a vector of transmission for the inoculation of pathogens from infected to healthy oral tissues. *Enterococcus faecalis* (*Ef*) is a gram-positive bacterium associated with cardiac/gastrointestinal infection and antibiotic resistance, and because of it is important to control in TBs disinfection and periodic replacement [12, 14, 26].

The maintenance of TB hygiene is essential in the daily practice of oral hygiene at home [17], and thus, accessible techniques that act against pathogens are required. Therefore, the aim of the study was to determine the efficacy of in vitro study of household techniques (CHX, WV, MW, and garlic alcohol extract [GARLIC]) for the disinfection of TBs contaminated with *Ef*. The null hypothesis was that household techniques are not effective for disinfecting TBs contaminated with this microorganism.

## 2. Materials and Methods

### 2.1. Study Design

This experimental in vitro study was conducted by the Stomatology Career and the Biochemistry and Microbiology laboratories of the Universidad Científica del Sur and approved with registration no. 0028-DACE-DAFCS-U.CIENTÍFICA−2023 and developed according to the CRIS—Checklist for Reporting In vitro Studies (Table S1) [28].

### 2.2. Experimental Groups

The unit of analysis was TBs, and the unit of observation was the *Ef* ATCC 29212. The sample consisted of 76 TBs of standard dimensions contaminated with *Ef*. The inclusion criteria were new TBs of the same model and brand (Colgate Palmolive Corp. twister, Peru). The sample was randomly assigned into six groups: two negative controls of eight samples each (distilled water [DW], 10% dimethylsulfoxide [DMSO]), one positive control of 15 samples (0.12% CHX), and three groups of disinfection techniques of 15 samples each (100% WV, MW at 700 W, GARLIC 200 mg/mL). The sample size was calculated using Epidat v.4.2 software. Based on the results of a pilot study using the formula for comparison of means of colony-forming units per millimeter (CFU/mL) where the difference was 2251.8 (deviation: GARLIC = 2828.43 and MW = 577.35); 95% confidence level; and a power of 80%, giving 15 per group.

### 2.3. Alcoholic Extract of *Allium sativum* “Garlic”

Two kilograms of garlic were purchased. The garlic was of the variety “Morado Arequipeño” (Arequipa 2200 m.a.s.l.) and identified as the species *A. sativum L*. by el Herbario del Museo de Historia Natural of the Universidad Nacional Mayor de San Marcos (N°100-USM-MHN−2022). The selection and classification of garlic was carried out considering the size of of medium bulbs (5 cm), fresh (not refrigerated), well preserved (“not burst”, nor decomposed). The garlic cloves were washed with DW, drained, sliced and cooled to −20°C × 10 min and then frozen at −80°C × 24 h. The garlic slices were then freeze-dried to obtain bioactive compounds at −105°C × 0.010 mbar × 24 h. A subsequent grinding with mortar and pylon helped to obtain fine particles that were immersed in cane alcohol and DW at the proportion of 5 g garlic: 72 mL alcohol: 8 mL DW, and then sonicated at 40°C × 12,000 rpm × 20 min. The filtration was carried out with a vacuum system with Whatman N°2 paper and then the solvent was removed by means of a rotary steam at 40°C × 18 rpm × −1.0 bar. The concentrate obtained was placed in an oven at 40°C, and the solid residue was stored at −20°C [29, 30]. Finally, the garlic resin was diluted in 10% DMSO at a concentration of 200 mg/mL and then sterilized through a 0.22 μm filter [18] (Figure S1). The process was carried out in the Biochemistry Area by a chemical engineer (O.R.).

### 2.4. Inhibitory and Bactericidal Concentrations

A quantitative microdilution test of the sample was performed to determine the lowest concentration capable of inhibiting the growth of *Ef* in sterile 96-well microplates. Previously, the concentration of bacteria was determined using the McFarland scale 0.5 (1.5 × 10^8^ CFU/mL) and adjusted to a concentration of 1.5 × 10^5^ CFU/mL. The inoculum added to the culture medium was brain-heart infusion broth (BHI), which was subjected to different concentrations of GARLIC and was incubated at 37°C × 24 h to determine the minimum inhibitory concentration (MIC) [31]. Then, 100 μL of resazurin was added to the inoculum to define bacterial growth by observing pigmentation in blue tones (negative) and pigmentation in reddish tones (positive). Subsequently, the dissemination method for CFU was performed by seeding dilutions of the order to determine the minimum bactericidal concentration (MBC) in the Microbiology Department with the help of a biologist (R.O.M.).

### 2.5. Contamination and Disinfection of TBs

The TB heads were immersed in the bacterial suspension of *Ef* at a concentration of 1.5 × 108 CFU/mL in BHI broth for 5 min [10, 19, 20]. The heads were then randomly immersed individually for a period of 2 h in sterile plastic containers containing 3 mL of the disinfection liquids, which according to the groups were labeled as 0.12% CHX, 100% WV, GARLIC (200 mg/mL), 10% DMSO, and DW. Another group of TBs was placed in a sterile beaker for individual disinfection in the MW group at 700 W × 3 min [10] (Table 1).

### 2.6. Seeding and Incubation

**Figure 1:**
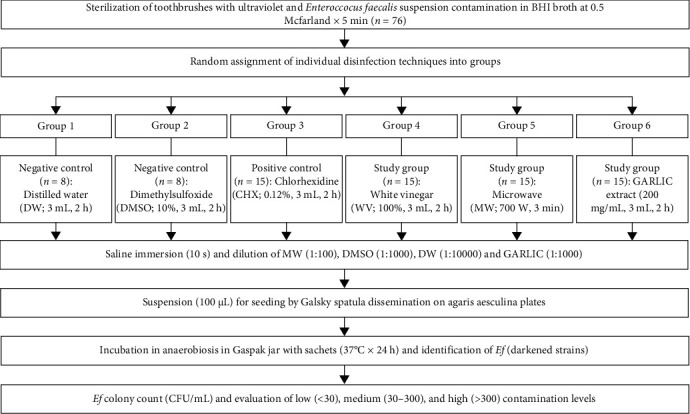
Flowchart of the TB disinfection experiment. TB, toothbrush.

The heads of the previously disinfected TBs were introduced in sterile physiological saline solution for 10 s and then shaken to remove the microorganisms. From this new suspension, dilutions were performed in the MW (1 : 100); DMSO (1 : 1000); DW (1 : 10,000); and GARLIC (1 : 1000) groups, with no dilutions in the CHX and WV groups. Subsequently, a 100 μL suspension was extracted and seeded with the dissemination method on esculin bile agar plates and incubated at 37°C × 24 h [21] under anaerobic conditions inside a GasPak jar with anaerobic sachets to differentiate *Ef*. Lastly, the *Ef* were measured in CFU/mL and categorized according to contamination: low (<30 CFU/mL), medium (30–300 CFU/mL), and high (>300 CFU/mL) [25] (Figure S2). The Figure 1 shows a flow chart of the study procedure.

### 2.7. Data Analysis

The descriptive statistics used were the mean, standard deviation, median, interquartile range (IQR), quartile 1 and 3. The CFU/mL variable was evaluated in the normal distribution with the Shapiro–Wilk test (*p* < 0.05) and for homoscedasticity using Levene's test (*p* < 0.05). The data were statistically analyzed using the nonparametric Kruskal–Wallis test with post hoc analysis in pairs. The data were analyzed using SPSS version 26 (SPSS inc., Armonk, NY, United States) at a significance level of *p* < 0.05.

## 3. Results

The concentration of the GARLIC that was determined in the MIC and MBC tests was 200 mg/mL, since in the plate sowing there was no bacterial growth at this concentration. Bacterial count (CFU/mL) of *Ef* in the disinfection groups are presented in Table 2. The results of the post hoc analysis in pairs are shown in Table 3. The effect of the different disinfection techniques on *Ef* showed highly significant differences between groups (*p* < 0.001), with lower bacterial counts (CFU/mL) in the CHX and WV groups (Me = 0; IQR = 0), and MW (Me = 6000; IQR = 45,000) versus both negative control groups (Me = 378,500 to 5,020,000; *p* < 0.001, *p* < 0.001, and *p* < 0.019, respectively). The counts of the MW group were not statistically different than CHX (*p*=0.06), WV (*p*=0.06), and the GARLIC groups (*p*=0.822). The antibacterial effect of the GARLIC [Me = 1,510,000; IQR = 590,000] was inferior to CHX and WV (*p* < 0.001), but similar to both negative control groups (*p* > 0.999).

**Figure 2:**
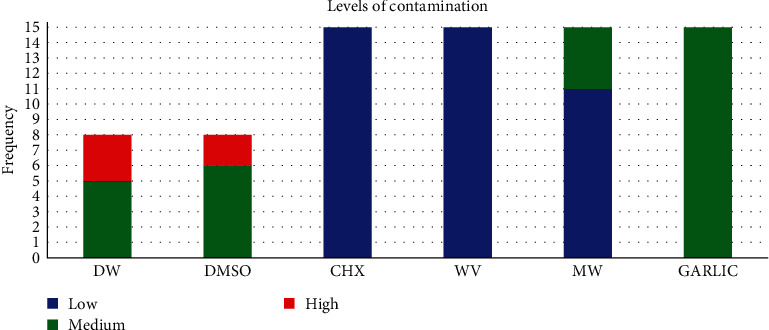
Level of *Ef* contamination in the different disinfection groups. CHX, Chlorhexidine; DMSO, dimethylsulfoxide; DW, distilled water; *Ef*, *Enteroccocus faecalis*; GARLIC, garlic alcohol extract, MW, microwave; WV, white vinegar.

Figure 2 shows the level of *Ef* contamination in the different disinfection groups. The level of *Ef* contamination was low in all plates of the CHX and WV groups (*n* = 15; 100%) and in most of the MW plates (*n* = 11; 73.3%), being medium in all the GARLIC plates (*n* = 15; 100%). The level of contamination of the plates in the DMSO and DW groups was medium (75% and 62.5%, respectively) and high (25% and 37.5%, respectively).

## 4. Discussion

There are cultural and economic barriers unique to each person that require the search for simple and accessible methods for disinfecting TBs [18]. This study evaluated four methods of household disinfection that can be found in Peruvian homes, a rinse-type chemical agent (CHX), a natural culinary agent (WV), a radiation agent (WV), and a natural agent converted to extract (GARLIC). The hypothesis was tested by finding which CHX, WV, and MW domestic techniques were effective for the disinfection of TBs contaminated with *Ef*.

This study simulated a situation of TB utilization. The DW and DMSO control groups did not decontaminate the TBs of *Ef*. DMSO is considered as an organic solvent and was used in the preparation of the garlic extract, which showed negative results in the control of *Ef*, that shows it did not influence the results of the GARLIC group. While we sought to improve the condition of a habitual flushing of TB with tap water with the use of DW, this was not sufficient and therefore, more effective methods are required [10, 22].

CHX is a broad-spectrum cationic agent considered to be gold standard biofilm control by generating cell wall lysis [23]. However, a previous review of 12 studies with different designs did not find a significant microbial reduction of *Ef* with CHX in TBs regardless of the concentration [26]. This contradicts the findings of this study in which CHX at 0.12% per 2 h eliminated *Ef* from TBs. Its efficacy in other microorganisms was also corroborated with 0.12% CHX [8, 20, 24] and CHX at 0.2% for 10 min [15], 1 h [22], and 12 h [23]. Currently, CHX is being increasingly accepted as an oral rinse in Peru considering its mass marketing in pharmacies and supermarkets, and thus, the expansion of the use of CHX to the maintenance of TBs that are still functional could balance the cost/benefit of TB replacement.

WV contains acetic acid that diffuses through the bacterial cell wall until it disintegrates [21]. This disinfectant property in TB immersed in WV for 2 h eliminated the *Ef* in the present study with results equal to CHX [16, 17, 21]. Other studies also found that WV generated a significant reduction of *Ef* of between 30% and 53% at concentrations of 70% and 100% [16] or was comparatively better compared to the control group (tap water or sodium chloride) at concentrations of 38% per 12 h [21] and 50% [17] and 100% [10] for 10 min for control of *Ef* [10] or cultured oral bacteria [16, 17, 21]. This method would be considered cost-effective, easily accessible, and appropriate for domestic use [17, 26]. However, the effects of residual taste on TB need to be analyzed [10].

The MW is an appliance commonly found in homes. Its thermal mechanism denatures intracellular proteins, causing bacterial cell lysis. This study showed that 700 W of MW for 3 min also had a statistically similar disinfectant effect as CHX and WV [16]. Other studies also reported its efficacy at times of 1 min ×1400 W [25], 3 min per 650 W, [10] and 5 min [16], and 7 min per 1100 W [20, 24], showing a significant reduction of oral bacteria cultures to more than half [16] or compared to groups with [10] or without [25] washing with tap water in the assessment of *Ef*. Although MW did not completely eradicate *Ef* growth, it did show a trend towards low levels of *Ef* contamination [25]. In addition, after 5 min in the MW, the TB was no longer suitable for use.

Garlic is a natural species composed of allicin that makes a chemical reaction with thiol groups affecting the essential metabolism of cysteine proteinase involved in the pathogenicity of oral microorganisms [23, 29]. This study showed that garlic had limited antimicrobial capacity with results statistically similar to negative control groups. The results were contradictory to those obtained in previous studies in which garlic, at 4.15 mg/mL [23] or in 3% extract [18], proved to be a highly effective antimicrobial solution with a 96% reduction in 96% [23] and 100% [18] in other types of bacteria, such as *Streptococcus mutans*, on TBs [18]. This difference in results was probably due to the way the extract was prepared, as in another previous study it was shown to be similar to sodium hypochlorite for the disinfection of dental canals [32].

The limitations of the study were to find several disinfection times from 5 min [19, 25], 12 h [21, 23, 26], and 24 h [23], but in this study we opted for a time of 3 min with MW and 2 h for the rest of the groups. Based on previous tests of disinfection of TBs with garlic, unlike other studies, the present study found limited antimicrobial efficacy, and therefore, it is recommended to adjust the processing and evaluate the mechanisms of its active components.

## 5. Conclusion

Within the limitations of this in vitro study, it was concluded that disinfection of TBs with CHX, WV, and MWs produces a significant effective reduction in the count of *Ef*, while GARLIC showed limited results in this reduction.

## Figures and Tables

**Table 1:** Distribution of disinfection techniques.

Group	Disinfection techniques	Brand, manufacture, lot	Concentration/potency	Time (min)	*n*
1	DW (negative control)	Droguería Criofarma, Peru, 2032043	—	120	8
2	DMSO (negative control)	CDH, India, 201114	10%	120	8
3	CHX (positive control)	Dentaid, Spain, 1005800	0.12%	120	15
4	WV	Florida, Peru, 7751158005913	100%/acidity 5%	120	15
5	MW	Samsung, China	700 W	3	15
6	GARLIC	Arequipa, Perú, *A. sativum L*	200 mg/mL	120	15
Total					76

Abbreviations: CHX, chlorhexidine; DMSO, dimethylsulfoxide; DW, distilled water; GARLIC, alcoholic extract of garlic; MW, microwave; WV, white vinegar.

**Table 2:** Bacterial count (CFU/mL) of *Ef* in the disinfection groups.

Disinfection techniques	CFU		
	Media ± SD	Median [Q1–Q3]	*p* value
DW	5,296,250.00 ± 3,019,862.52	3,785,000 [3,095,000–7,700,000]^a^	<0.001*⁣*^*∗*^
DMSO	6,345,000.00 ± 3,746,034.09	5,020,000 [3,090,000–9,850,000]^a^	
CHX	0 ± 0	0 [0–0]^c^	
WV	0 ± 0	0 [0–0]^c^	
MW	32,000 ± 55,532.49	6000 [2000–47,000]^bc^	
GARLIC	1,531,333.33 ± 335,577.68	1,510,000 [1,210,000–1,800,000]^ab^	

*Note:* Kruskal–Wallis test.

Abbreviations: CFU, colony forming units; CHX, chlorhexidine; DMSO, dimethylsulfoxide; DW, distilled water; GARLIC, alcoholic extract of garlic; MW, microwave; WV, white vinegar.

⁣^*∗*^*p*  < 0.001.

**Table 3:** Comparison of bacterial counts (CFU/mL) of *Ef* in the disinfection groups.

Disinfection techniques	*p* value				
	DMSO	CHX	WV	MW	GARLIC
DW	1.000	<0.001*⁣*^*∗∗*^	<0.001*⁣*^*∗∗*^	0.019*⁣*^*∗*^	>0.999
DMSO	—	<0.001*⁣*^*∗∗*^	<0.001*⁣*^*∗∗*^	0.015*⁣*^*∗*^	>0.999
CHX	—	—	>0.999	0.060	<0.001*⁣*^*∗∗*^
WV	—	—	—	0.060	<0.001*⁣*^*∗∗*^
MW	—	—	—	—	0.822

*Note:* Kruskal–Wallis test with post hoc analysis in pairs.

Abbreviations: CFU, colony forming units; CHX, chlorhexidine; DMSO, dimethylsulfoxide; DW, distilled water; GARLIC, garlic alcohol extract; MW, microwave; WV, white vinegar.

⁣^*∗*^*p*  < 0.05; ⁣^*∗∗*^*p*  < 0.001.

## Data Availability

The data obtained to support the results of this study are available at https://figshare.com/s/c3eead2509b3b171724b.

## Supporting Information

Supporting Information Consolidated appendix. Table S1: CRIS (Checklist for Reporting In vitro Studies). Figure S1: garlic extract production process. Figure S2: procedures of the experiment.
