# Supplementary material for: In Vitro Efficacy of Domestic Techniques for Disinfection of Toothbrushes Contaminated With Enterococcus faecalis
Source: Int J Dent. 2024 Oct 18;2024:3509832. doi: 10.1155/2024/3509832 (PMC11511586; doi:10.1155/2024/3509832)
Supplement: Supporting Information — Consolidated appendix. Table S1: CRIS (Checklist for Reporting In vitro Studies). Figure S1: garlic extract production process. Figure S2: procedures of the experiment. [file 3509832.f1.docx]

Supplementary Materials

**Table S1.** CRIS (Checklist for Reporting *In-vitro* Studies).

| Section/Topic | Item  Nº | Checklist item | Reported on page No |
| --- | --- | --- | --- |
| Title and abstract |  |  |  |
|  | 1a | Identification as an in vitro/laboratory study in the title | 1 |
|  | 1b | Structured summary of trial design, methods, results, and conclusions | 1 |
| **Introduction** | 2a | Scientific background and explanation of rationale | 2 |
| Background and objectives | 2b | Specific objectives or hypotheses | 2 |
| **Methods** |  |  |  |
| Interventions | 3 | The intervention for each group, including how and when they were actually administered, with sufficient detail to allow replication | 3-4 |
| Outcomes | 4 | Completely defined pre-specified primary and secondary outcome measures, including how and when they were assessed | 3-6  Figure S1  Figure S2 |
| Sample size | 5 | How sample size was determined | 3 |
| Randomisation: Sequence generation | 6 | Method used to generate the random allocation sequence | Random assignment to exposure groups  3 |
| Allocation concealment mechanism | 7 | Mechanism used to implement the random allocation sequence (such as sequentially numbered containers), describing any steps taken to conceal the sequence until interventions were assigned | Figure 1 |
| Implementation | 8 | Who generated the random allocation sequence, who enrolled teeth, and who assigned teeth to intervention | NA |
| Blinding | 9 | If done, who was blinded after assignment to interventions (for example, care providers, those assessing outcomes) and how outcome) | NA |
| Statistical methods | 10 | Statistical methods used to compare groups for primary and secondary outcomes | 7 |
| Results |  |  |  |
| Numbers analysed | 11a | For each group, number of ‘items’ (drugs) included in each analysis and whether the analysis was by original assigned groups | 7-8  Table 2  Table 3  Figure 2 |
| Outcomes and estimation | 11b | For each primary and secondary outcome, results for each group, and the estimated effect size and its precision (such as 95% confidence interval) | 7-8 |
| **Discussion** |  |  |  |
| Limitations | 12a | Trial limitations, addressing sources of potential bias, imprecision, and, if relevant, multiplicity of analyses | 10 |
| Generalisability | 12b | Generalisability (external validity, applicability) of the trial findings | 9 |
| Interpretation | 12c | Interpretation consistent with results, balancing benefits and harms, and considering other relevant evidence | 9-10 |
| Other information |  |  |  |
| Protocol | 24 | Where the full trial protocol can be accessed, if available | NA |
| Funding | 25 | Sources of funding and other support (such as supply of drugs), role of funders | 11 |

*Krithikadatta J, Gopikrishna V, Datta M. CRIS Guidelines (Checklist for Reporting In-vitro Studies): A concept note on the need for standardized guidelines for improving quality and transparency in reporting in-vitro studies in experimental dental research. J Conserv Dent. 2014;17(4):301–304.

| 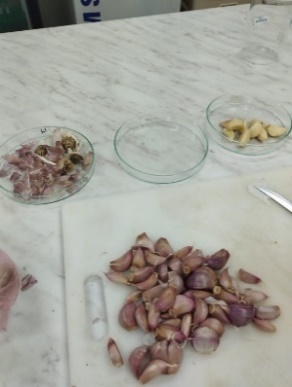  **A** | 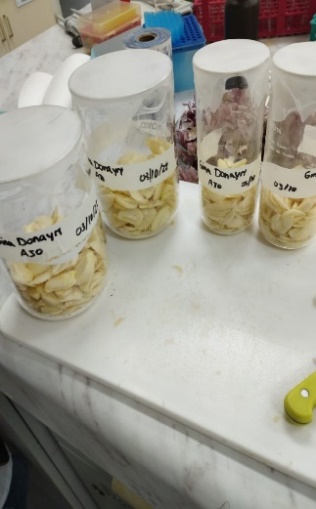  **B** | 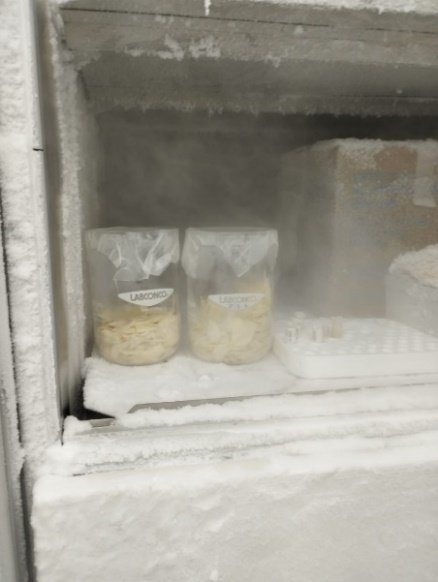  **C** |
| --- | --- | --- |
| 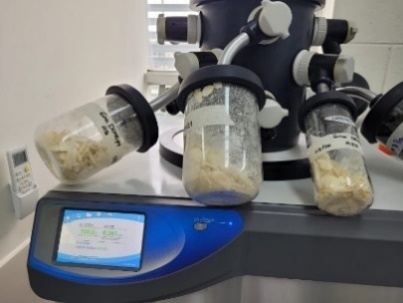  **D** | 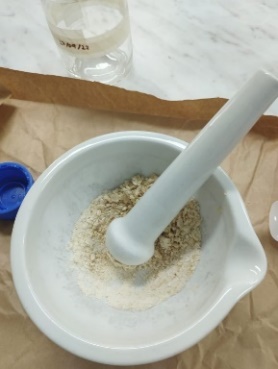  **J**  **K**  **GA**  **L**  **E** | 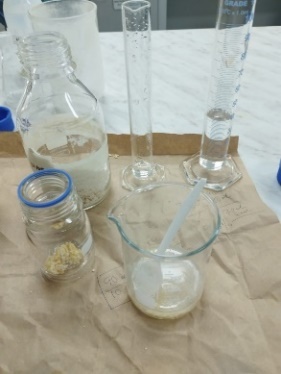  **F** |
| 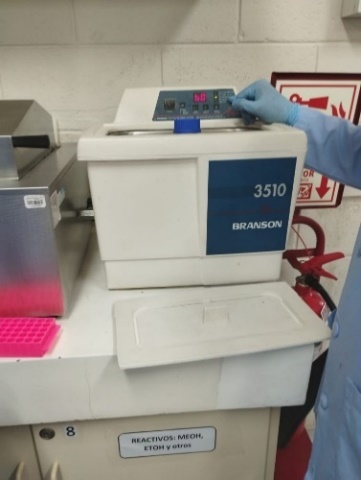  **H** | 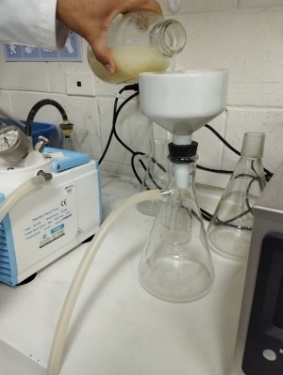  **I** | 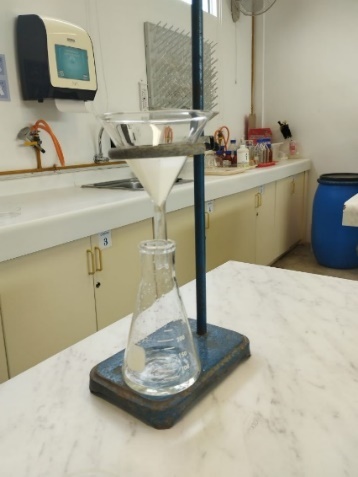 |
| 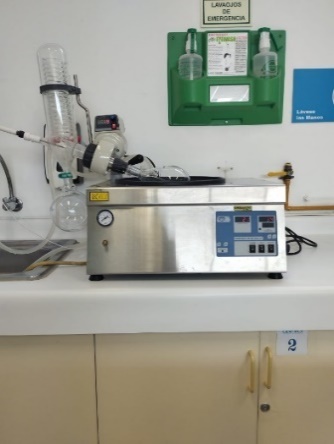 | 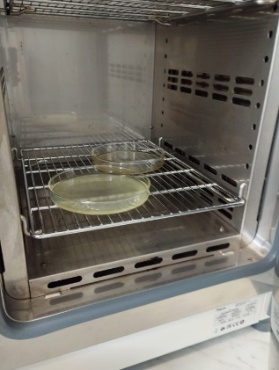 | 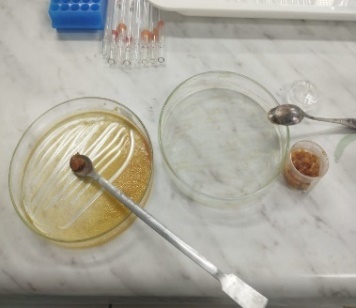 |
| 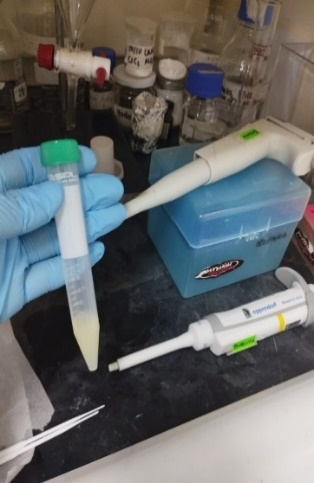  **M** | 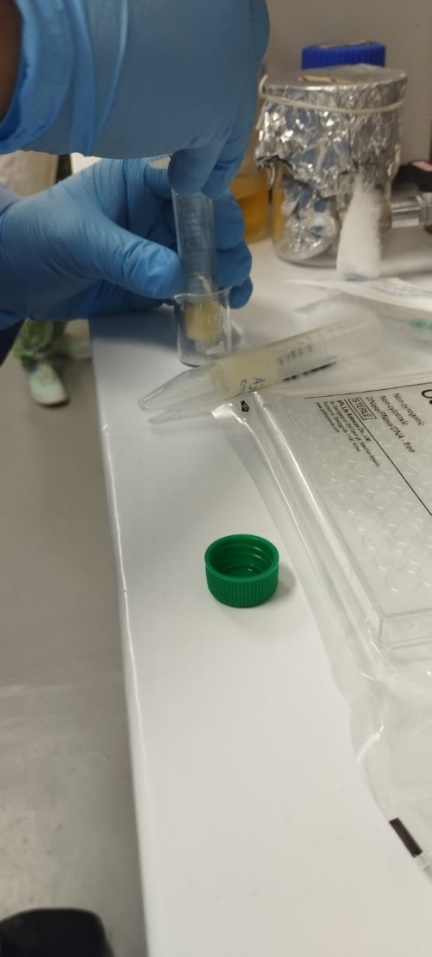   | 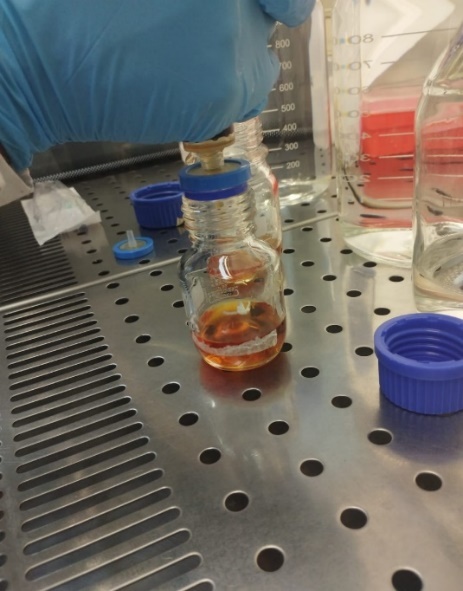  **O** |

**Figure S1.** Garlic extract production process: (A) Selection and peeling of garlic cloves, (B) Slicing, (C) Frozen at -80°C, (D) Freeze-dried x 24 h, (E) Grinding with mortar and pylon, (F) Alcoholic extraction, (G) Sonication x 1 h, (H) Vacuum filtration (I) Conventional filtration (J) Partial removal of solvent with rotary steam (K) Total removal of solvent with stove at 40 °C (L) Obtaining solid residue ( M) Dilution in 10% DMSO (N) Third filtrate (O) Sterilization of the extract with a 0.22 um filter.

| 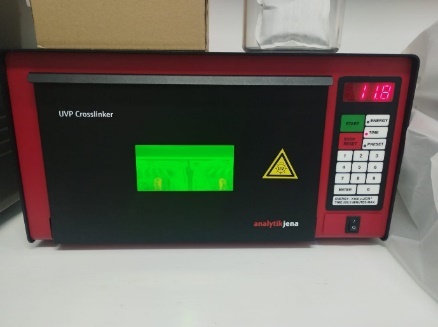  **B**  **A** | 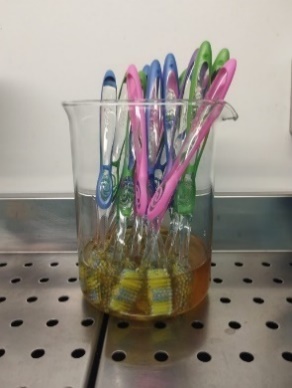  **C** | 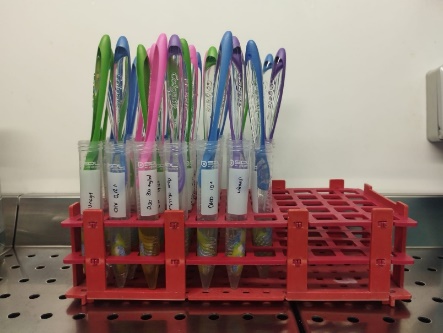 |
| --- | --- | --- |
| 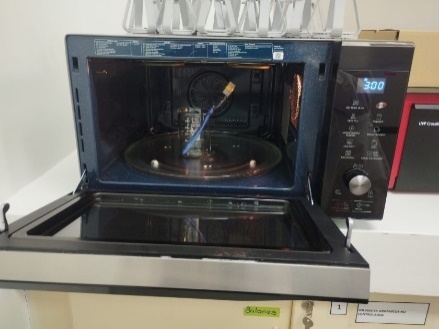  **E** | 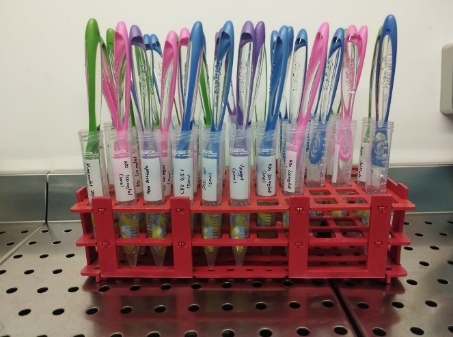 | 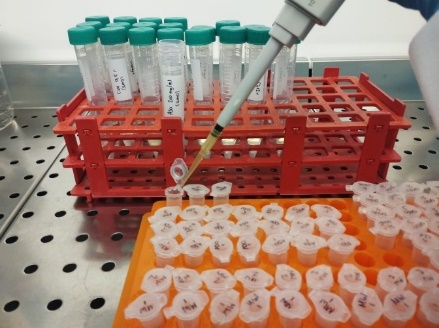  **F** |
| 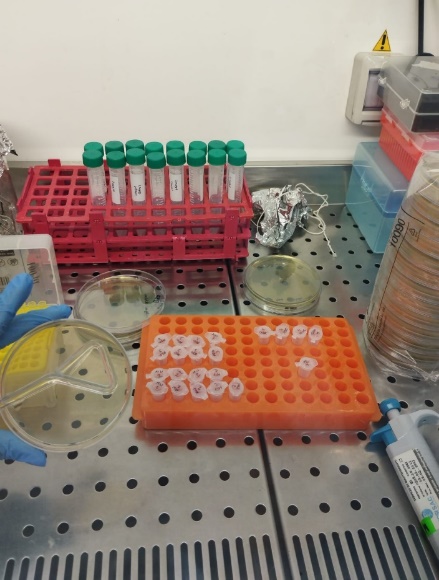 | 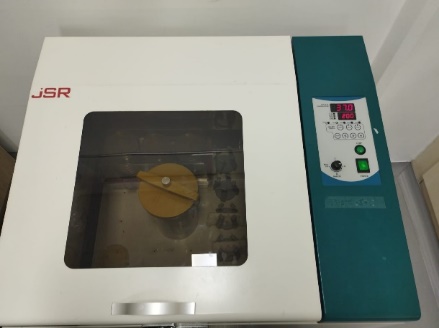  **H** | 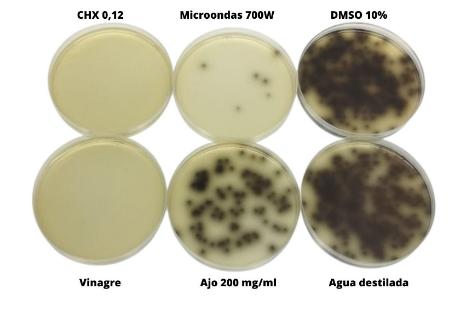  **I** |

**Figure S2.** Procedures of the experiment: (A) Sterilization of brushes with ultraviolet rays, (B) Contamination of toothbrushes with *Ef*, (C) Disinfection of toothbrushes in liquids, (D) Disinfection of toothbrushes in microwaves, (E) Immersion in sterile saline solution, (F) Serial dilutions, (G) Seeding in agaris aculin bile, (H) Incubation, (I) Comparison of plates obtained.

**D**

**G**
